# Monitoring tumor response to neoadjuvant chemotherapy using MRI and ^18^F-FDG PET/CT in breast cancer subtypes

**DOI:** 10.1371/journal.pone.0176782

**Published:** 2017-05-22

**Authors:** Alexander M. Th. Schmitz, Suzana C. Teixeira, Kenneth E. Pengel, Claudette E. Loo, Wouter V. Vogel, Jelle Wesseling, Emiel J. Th. Rutgers, Renato A. Valdés Olmos, Gabe S. Sonke, Sjoerd Rodenhuis, Marie Jeanne T. F. D. Vrancken Peeters, Kenneth G. A. Gilhuijs

**Affiliations:** 1 Department of Radiology, The Netherlands Cancer Institute, Amsterdam, Netherlands; 2 Department of Radiology / Image Sciences Institute; University Medical Center Utrecht, Utrecht, Netherlands; 3 Department of Nuclear Medicine, The Netherlands Cancer Institute, Amsterdam, Netherlands; 4 Department of Pathology, The Netherlands Cancer Institute, Amsterdam, Netherlands; 5 Department of Surgery, The Netherlands Cancer Institute, Amsterdam, Netherlands; 6 Department of Medical Oncology, The Netherlands Cancer Institute, Amsterdam, Netherlands; University of North Carolina at Chapel Hill School of Medicine, UNITED STATES

## Abstract

**Purpose:**

To explore guidelines on the use of MRI and PET/CT monitoring primary tumor response to neoadjuvant chemotherapy (NAC), taking breast cancer subtype into account.

**Materials and methods:**

In this prospective cohort study, 188 women were included with stages II and III breast cancer. MRI and ^18^F-FDG-PET/CT were acquired before and during NAC. Baseline pathology was assessed from tumor biopsy. Tumors were stratified into HER2-positive, ER-positive/HER2-negative (ER-positive), and ER-negative/PR-negative/HER2-negative (triple-negative) subtypes, and treated according to subtype. Primary endpoint was pathological complete response (pCRmic) defined as no or only small numbers of scattered invasive tumor cells. We evaluated imaging scenarios using MRI only, PET/CT only, and combinations.

**Results:**

pCRmic was found in 35/46 (76.1%) of HER2-positive, 11/87 (12.6%) of ER-positive, and 31/55 (56.4%) of triple-negative tumors. For HER2-positive tumors, MRI yielded the strongest predictor (AUC: 0.735; sensitivity 36.2%), outperforming PET/CT (AUC: 0.543; p = 0.04), and with comparable results to combined imaging (AUC: 0.708; p = 0.213). In ER-positive tumors, the combination of MRI and PET/CT was slightly superior (AUC: 0.818; sensitivity 55.8%) over MRI alone (AUC: 0.742; p = 0.117) and PET/CT alone (AUC: 0.791). However, even though relatively large numbers of ER-positive tumor patients were included, no significant differences were yet found. For triple-negative tumors, MRI (AUC: 0.855; sensitivity 45.4%), PET/CT (AUC: 0.844; p = 0.220) and combined imaging (AUC: 0.868; p = 0.213) yielded comparable results.

**Conclusions:**

For HER2-positive tumors, MRI shows significant advantage over PET/CT. For triple-negative tumors, comparable results were seen for MRI, PET/CT and combined imaging. For ER-positive tumors, combining MRI with PET/CT may result in optimal response monitoring, although not yet significantly.

## Introduction

Neoadjuvant chemotherapy (NAC) for breast cancer has the potential benefit of reducing tumor size, enabling conversion from mastectomy towards breast-conserving surgery [1–3] as well as reduction in the extent of axillary lymph node surgery [4–6]. In addition, the response to chemotherapy can be monitored; which enables switching to alternative non-cross resistant chemotherapy or ceasing treatment after insufficient response. Thus, patients may either benefit from a more appropriate NAC regimen or they will be protected from undergoing further ineffective toxic treatment [7].

Monitoring treatment response during NAC is typically performed using ultrasound or dynamic contrast-enhanced (DCE) magnetic resonance imaging (MRI). The latter has the potential to discriminate between viable tumor cells and NAC-induced fibrotic tissue and has shown to be a strong predictor for tumor response [8–10]. Although MRI has several advantages over conventional imaging techniques, the predictive value of MRI is not perfect and it strongly depends on the molecular subtype and morphologic appearances of tumors [11]. MRI performs well in human epidermal growth factor receptor 2 (HER2)-positive tumors, and in estrogen receptor (ER)-negative/progesterone receptor (PR)-negative/HER2-negative (triple-negative) tumors, but it is less accurate in ER-positive tumors [12].

Hence, other imaging techniques are under investigation to monitor tumor response [13]. Currently, positron emission tomography using fluorodeoxyglucose, integrated with computed tomography (^18^F-FDG PET/CT), is used for preoperative staging in patients scheduled for NAC [14]. Also it has been investigated to monitor response of breast cancer to NAC [15,16]. The results for PET/CT also showed dependence on breast cancer subtype, indicating good performance in ER-positive and triple negative tumors, but relatively poor performance in HER2-positive tumors [17].

MRI visualizes changes in morphology and vascularization of tumors whereas PET/CT visualizes changes in the glucose metabolism of tumors. Therefore, a complementary value of these techniques has been hypothesized. This complementary value for response monitoring is important knowing both imaging techniques vary in accuracy depending on breast cancer subtype. Recently, an explorative study showed a potential complementary value of MRI and PET/CT. However, this study had an insufficient number of patients to determine how MRI and PET/CT could be combined in the daily clinical workflow to benefit optimally from their complementary value [18].

The aim of the present study is to explore guidelines on the use of MRI and PET/CT in the clinical workflow to monitor response of the primary tumor to NAC, taking breast cancer subtype into consideration.

## Materials and methods

### Patient cohort

Patients were included between September 2008 and June 2013 in this prospective cohort study. Eligibility criteria included primary invasive breast cancer of at least 3 cm and/or at least one tumor-positive axillary lymph node. This study was approved by the institutional review board of the Netherlands Cancer Institute—Antoni van Leeuwenhoek hospital (METC AVL) in Amsterdam and written informed consent was obtained from all patients. Of this current study, 93 patients were reported earlier by Pengel et al. [18].

### Pathology prior to NAC

Core-needle biopsies of the primary tumor were taken prior to NAC. Tissue was routinely processed and stained using hematoxylin and eosin. Histopathology was assessed by an experienced breast pathologist (J.W.). Tumor type was recorded as invasive ductal carcinoma (IDC), invasive lobular carcinoma (ILC) or any ‘other’ tumor type. The estrogen receptor (ER), progesterone receptor (PR), and human epidermal growth factor receptor 2 (HER2) status were determined according to the Dutch guidelines (www.oncoline.nl). For ER and PR, immunohistochemistry was used. A 10% threshold was used to discriminate between negative (<10% staining) or positive (≥10% staining) hormone receptor status. Immunohistochemistry for the HER2 was scored as 0, 1+, 2+ or 3+ to differentiate between negative (<2+) and positive (>2+) HER2 receptor status. At score 2+, in-situ hybridization was used to differentiate between a negative and positive status. Tumors were stratified into ER-positive and HER2-negative subtype (ER-positive), HER2-positive subtype (HER2-positive) and ER-negative/PR-negative/HER2-negative (triple-negative) subtype.

#### NAC

The NAC regiment differed per subtype (18). In short, HER2-positive tumors were treated in three cycles of eight weeks with paclitaxel, carboplatin and trastuzumab (day 1, 8, 15, 22, 29 and 36) [19]. ER-positive and triple-negative tumors were treated with three courses of ddAC (doxorubicin and cyclophosphamide on day 1, every 14 days, with PEG-filgrastim on day 2). Following these three courses, tumors were reported as ‘favorable’ or ‘unfavorable’ responders based on previously reported MRI response criteria by Loo et al. (8). In the context of a larger study, a ‘favorable response’ was followed by three more courses of ddAC whereas an ‘unfavorable response’ was followed by three courses of docetaxel and capecitabine, which criteria were reported earlier by Rigter et al. [20].

### Response imaging

MRI and PET/CT were performed at the start of chemotherapy (baseline imaging) and during chemotherapy (interim imaging), specified as after the first cycle of eight weeks (in HER2-positive tumors) or after three courses of chemotherapy (in ER-positive and triple-negative tumors) [21].

### MRI

MRI was performed using a 3.0-T scanner (Achieva, Philips, Best, The Netherlands) with dedicated bilateral seven-element SENSE breast coil. Patients were scanned in prone orientation. Six consecutive coronal 3-D THRIVE SENSE T1-weighted sequences were acquired (1.1 x 1.1 x 1.1 mm^3^ voxels; 90s acquisition time; TR/TE 4.4/2.3 ms, flip angle 10°, FOV 360mm); One unenhanced series and five series following the intravenous injection (power injector; 3 mL/s) of gadolinium-containing contrast (Dotarem 0.5 mmol/ml; Guerbet; Aulnay-sous-Bois, France) which was followed by 30 mL of saline.

MR imaging was assessed by radiologists with breast MR experience using a protocol as previously described [8,22]. In short, a custom-built viewing station was used which enabled simultaneous viewing of two series reformatted and linked in three orthogonal directions. Subtraction images for initial enhancement (90s after contrast agent injection), late enhancement (450s after contrast agent injection), maximum intensity projections, and color-coded visualization of contrast curves were available. The latter visualized enhancement into persisting, plateau or a wash-out curve in accordance with the definitions used by Kuhl et al. [23]. The largest tumor diameter was assessed at initial (LD initial) and at late (LD late) enhancement. The largest diameter spanned the total lesion-bearing region including seemingly normal tissue in between and in any of the three orthogonal directions. Relative changes on MRI (MRI Δ) between interim and baseline imaging were calculated separately for LD initial and LD late.

### PET/CT

Imaging with PET/CT was performed after a six-hour fasting period at blood glucose levels of <10 mmol/l. Ten milligrams of diazepam were administered orally to prevent brown adipose tissue activation [24]. Depending on body mass index an intravenous dose of 180 or 240 MBq FDG was administered. After a resting period of 60 ± 10 min, PET/CT (Gemini TF; Philips, Cleveland, Ohio) was performed with the patient in prone orientation using a stripped mock-up MRI coil. The CT scan (10 mAs, 2mm slices) preceded the PET scan (3 min per bed position; 2 x 2 x 2 mm^3^ voxels). An additional standard supine whole-body PET/CT scan for distant staging was performed at baseline imaging prior to NAC. A panel of experienced readers evaluated the images in an orthogonal multiplanar reconstruction; which simultaneously display PET, CT, and fused PET/CT imaging. FDG uptake was measured using maximum standardized uptake values (SUV-max) in a 3D region of interest containing the primary tumor (SUV-max tumor) and, when present, in the lymph node (SUV-max lymph node) showing the strongest uptake [17]. Relative changes on PET/CT (PET/CT Δ) between interim and baseline imaging were calculated separately for the SUV-max tumor and the SUV-max lymph node.

### Pathology after NAC

In this study, according to the definition of Sataloff et al. [25], pathological complete response (pCRmic) after completion of NAC, was defined as either complete absence of tumor cells or presence of only a small number of scattered invasive cells in the breast resection specimen (ypTmic). Pathological non-complete response (non-pCRmic) was defined as any remaining viable residual disease in the breast due to partial tumor response, stable or progressive disease.

### Analyses

#### Baseline characteristics

Analyses were performed using SPSS (version 20.0; Chicago, Illinois). Associations were assessed between pCRmic and patient age, tumor histology, tumor subtype, MRI curve-type prior to NAC, MRI LD initial, MRI LD late, SUV-max tumor, SUV-max lymph node, as well as the change of these latter four characteristics during NAC. Two-sided Pearson’s chi squared, Fisher’s exact, and Mann-Whitney U tests were used for this purpose.

#### Imaging scenarios

**Fig 1:**
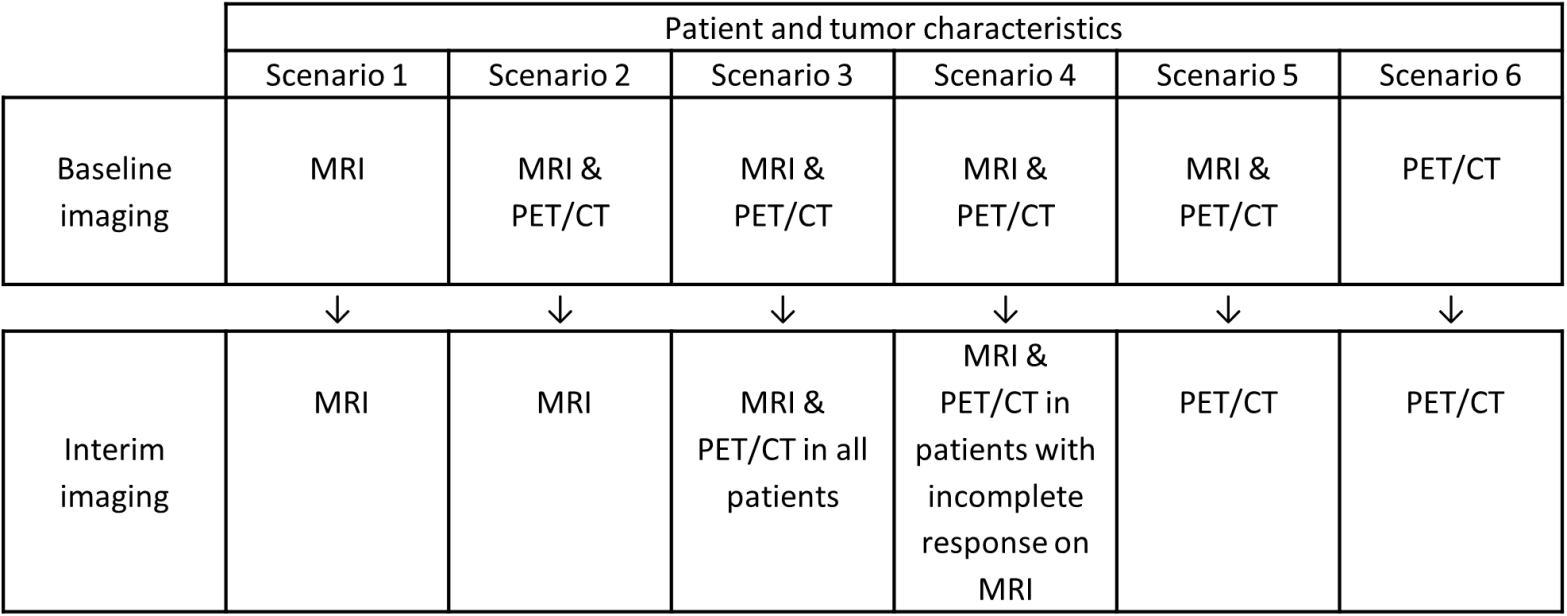
The six potential imaging scenarios investigated to monitor response of tumors during neoadjuvant chemotherapy.

At the interim-imaging stage, post-hoc analysis was performed to systematically evaluate and compare six different imaging scenarios for response monitoring per subtype: MRI only, PET/CT only, MRI and PET/CT at baseline with MRI only or PET/CT only at interim imaging, MRI followed by PET/CT, or MRI followed by PET/CT only under certain conditions (Fig 1). For every imaging scenario, the patient, tumor and scenario-specific imaging characteristics were entered into multivariate analyses (binary logistic regression with backward feature selection, p-to-remove: 0.10). Receiver operating characteristics (ROC) curves were acquired and areas under the curve (AUC) were assessed. Subsequently, patients were stratified according to breast cancer subtype. The AUC of the different scenarios were compared using the DeLong test [26]. For this purpose, the scenario to monitor response using MRI only was used as a reference. ROC-curves were fitted using bi-exponential fitting [27], and an operating point at 90% specificity was selected to assess the accompanying sensitivity. In other words, the probability of correctly predicting a non-pCRmic was determined under the condition that the probability to correctly predict a pCRmic is at least 90%.

## Results

### Baseline patient and pathology characteristics

A total of 188 patients were included (mean age 47 years, range 25–73 years), baseline characteristics are shown in Table 1. According to ypTmic, which was used as pCRmic in this current study, overall 77/188 of patients (41%) achieved a pCRmic and a non-pCRmic was seen in 111/188 of patients (59%). Patients with pCRmic were significantly (p<0.001) younger (mean age: 44 years) than patients with non-pCRmic (mean age: 50 years).

**Table 1:** Baseline patient and tumor characteristics prior to neoadjuvant chemotherapy (NAC). Patient and tumor characteristics of all 188 patients versus pathological complete response (pCRmic) after NAC. sd: Standard deviation. IDC = Invasive ductal carcinoma. ILC = Invasive lobular carcinoma. ER = Estrogen receptor. HER2 = Human epidermal growth factor receptor 2.

Characteristic	Overall	pCRmic	Non-pCRmic	p-value
**Total (%)**	188	77 (41%)	111 (59%)	
**Age (years), mean (sd)**	47 (11)	44 (11)	50 (10)	<0.001
**Tumor stage prior to NAC**	** **			
*T1*	20	11	9	0.391
*T2*	116	47	69	
*T3*	43	17	26	
*T4*	9	2	7	
**Nodal stage prior to NAC**	** **			
*N0*	40	15	25	0.862
*N1*	105	43	62	
*N2*	12	5	7	
*N3*	31	14	17	
**Histology**				
*IDC*	167	72	95	0.222
*ILC*	18	4	14	
*Other*	3	1	2	
**Clinical Subtype, n (%)**				
*ER-positive/HER2-negative (%)*	87	11 (12.6%)	76 (87.4%)	<0.001
*HER2-positive (%)*	46	35 (76.1%)	11 (23.9%)	
*Triple negative (%)*	55	31 (56.4%)	24 (43.6%)	

Considering subgroups, for the ER-positive subgroup a pCRmic was seen in 11/87 patients (12.6%). Conversely, pCRmic was seen in 35/46 patients (76.1%) with HER2-positive tumors and in 31/55 patients (56.4%) with triple-negative tumors. No residual disease in the breast or axilla (ypT0/is ypN0) was seen in 26/46 (56.2%) of HER2-positive tumors, 4/87 (4.6%) of ER-positive tumors, and 22/54 (40%) of triple-negative tumors.

### Baseline imaging

On baseline MRI, the mean tumor size was 47 mm (LD initial) and 39 mm (LD late) (Table 2). No significant differences in size were observed between tumors where pCRmic was attained versus non-pCRmic. On baseline PET/CT, a significant difference was found between SUV-max in the tumor and response at pathology; tumors resulting in pCRmic had higher SUV-max (10.3) compared to those not leading to pCRmic (8.2) (p = 0.029). In addition, baseline SUV-max in the lymph nodes was higher in tumors resulting in pCRmic (5.7) than in those resulting in non-pCRmic (4.5), although this was not significant in the overall patient group (p = 0.056).

**Table 2:** Imaging characteristics prior to neoadjuvant chemotherapy. Imaging characteristics at MRI and PET/CT plotted versus the pathological complete response (pCRmic) and non-pCRmic of tumors to neoadjuvant chemotherapy. LD initial = Largest tumor diameter on initial enhancement. LD late = Largest tumor diameter on late enhancement. SUV-max = Maximum standardized uptake value.

Characteristic	Overall	pCRmic	Non-pCRmic	p-value
**MRI baseline; curve type**				0.212
*Persisting*	0	0	0	
*Plateau*	81	30	51	
*Wash-out*	107	47	60	
**MRI baseline; tumor size**				
*LD initial (mm); mean (sd)*	47 (24)	46 (23)	47 (25)	0.691
*LD late (mm); mean (sd)*	39 (21)	38 (18)	40 (22)	0.608
**PET/CT baseline; SUV-max**				
*SUV-max tumor; mean (sd)*	9.1 (6.0)	10.3 (7.2)	8.2 (4.7)	0.029
*SUV-max lymph node; mean (sd)*	5.0 (5.1)	5.7 (5.3)	4.5 (4.8)	0.056

### Interim imaging

**Fig 2:**
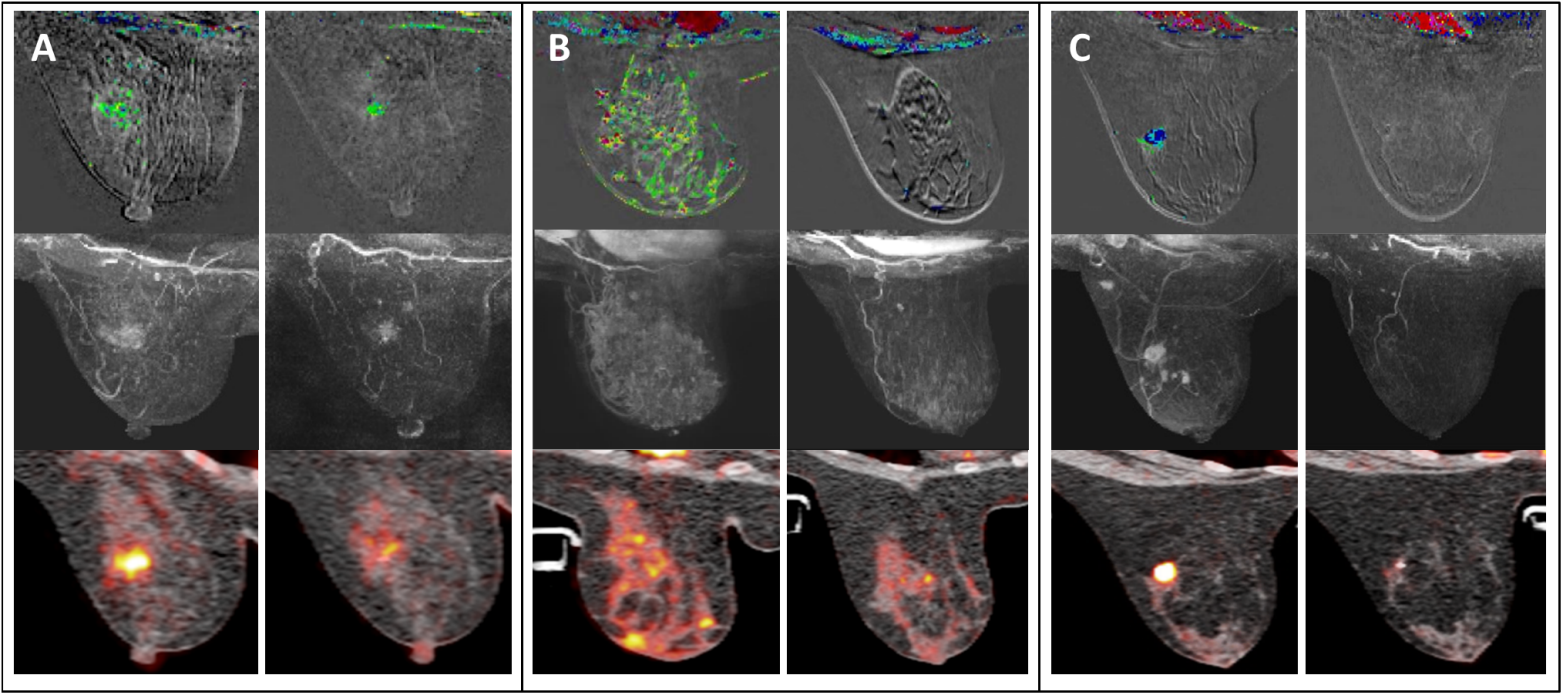
MRI and PET/CT imaging of different breast cancer subtypes. The top row shows MR subtraction images with color-coded visualization of contrast curves (persisting/green; plateau/blue; wash-out/red), the middle row shows maximum intensity projection of MR subtraction imaging, and the bottom row shows standardized uptake values on PET/CT imaging. For each example, imaging prior (left) and during (right) neoadjuvant chemotherapy is shown. (A) A 48-year-old women with an ER-positive invasive ductal carcinoma (IDC) showing a moderate response on MRI and PET/CT imaging, with a non-pathologic complete response (non-pCRmic) on final pathology. (B) A 52-year-old woman with a HER2-positive IDC showing a good response on MRI but a moderate response on PET/CT imaging, with a pCRmic on final pathology. (C) A 28-year-old woman with a triple-negative IDC showing a good response on MRI and PET/CT, with a pCRmic at final pathology.

During NAC, the relative change in size of tumors on MRI that reached pCRmic after NAC was significantly larger than the change in those that did not reach pCRmic (p<0.001) (Table 3). This was observed at initial enhancement (-66% change versus -26% change) as well as at late enhancement (-82% versus -42%) (Table 3). On PET/CT, the relative change in SUV-max of tumors resulting in pCRmic after NAC versus those resulting in non-pCRmic was significantly larger (-67% versus -43%; p<0.001). A comparable observation was made for changes in SUV-max in the lymph nodes (-74% versus -57%; p = 0.001). Examples of MRI and PET/CT imaging are shown in Fig 2.

**Table 3:** Imaging characteristics during NAC. Relative change (Δ) of largest tumor diameter on initial (LD initial) and late (LD late) enhancement on MRI ([LD interim–LD baseline / LD baseline] x 100%) and relative change of the maximum standardized uptake value (SUV-max) on PET/CT ([SUV-max interim–SUV-max baseline / SUV-max baseline x 100%] plotted versus pathological complete response (pCRmic) after NAC.

Characteristic	Overall	pCRmic	Non-pCRmic	p-value
**MRI Δ**				
*LD initial Δ (%); median (sd)*	-42 (34)	-66 (33)	-26 (24)	<0.001
*LD late Δ(%); median (sd)*	-58 (38)	-82 (28)	-42 (35)	<0.001
**PET/CT Δ**				
*SUV-max tumor Δ (%); median (sd)*	-53 (27)	-67 (17)	-43 (28)	<0.001
*SUV-max lymph node Δ (%); median (sd)*	-65 (32)	-74 (31)	-57 (31)	0.001

### Scenarios

An overview of the optimal model per scenario is given in Table 4. At interim imaging, the models resulting from scenarios 1 and 2 are identical, suggesting that baseline information from PET/CT does not add value to response monitoring without interim PET/CT. Comparable observations were found for scenarios 5 and 6: without interim MRI, baseline MRI does not add complementary information.

**Table 4:** Characteristics remaining in the scenario models. Characteristics remaining in scenario 1 to 6, with corresponding odds ratios (OR) and 95% confidence intervals (CI). B = Baseline imaging. I = Interim imaging. LD initial = Largest tumor diameter on initial enhancement. LD late = Largest tumor diameter on late enhancement. SUV-max = Maximum standardized uptake value. Δ = Relative change.

	Characteristics	OR	95% CI
*Scenario 1*	Age	0.961	0.925–0.998
*B*: *MRI*	Clinical subtype		
*I*: *MRI*	*Triple-negative*	*reference*	
	*ER-positive*	0.150	0.056–0.402
	*HER2-positive*	1.147	0.401–3.282
	LD initial Δ	0.171	0.030–0.975
	LD late Δ	0.126	0.027–0.580
*Scenario 2*	Age	0.961	0.925–0.998
*B*: *MRI & PET/CT*	Clinical subtype		
*I*: *MRI*	*Triple-negative*	*reference*	
	*ER-positive*	0.150	0.056–0.402
	*HER2-positive*	1.147	0.401–3.282
	LD initial Δ	0.171	0.030–0.975
	LD late Δ	0.126	0.027–0.580
*Scenario 3*	Clinical subtype		
*B*: *MRI & PET/CT*	*Triple-negative*	*reference*	
*I*: *MRI & PET/CT*	*ER-positive*	0.200	0.063–0.633
*in all patients*	*HER2-positive*	2.208	0.607–8.028
	SUV-max tumor Δ	0.032	0.003–0.359
	LD late MRI	0.100	0.023–0.434
*Scenario 4*	Clinical subtype		
*B*: *MRI & PET/CT*	*Triple-negative*	*reference*	
*I*: *MRI & PET/CT*	*ER-positive*	0.235	0.069–0.803
*in patients with*	*HER2-positive*	3.277	0.689–15.592
*incomplete*	LD late Δ	0.155	0.030–0.801
*response on MRI*	SUV-max tumor Δ	0.017	0.001–0.324
*Scenario 5*	Age	0.961	0.918–1.006
*B*: *MRI & PET/CT*	Clinical subtype		
*I*: *PET/CT*	*Triple-negative*	*reference*	
	*ER-positive*	0.256	0.089–0.740
	*HER2-positive*	4.902	1.484–16.195
	SUV-max tumor Δ	0.017	0.002–0.157
*Scenario 6*	Age	0.961	0.918–1.006
*B*: *PET/CT*	Clinical subtype		
*I*: *PET/CT*	*Triple-negative*	*reference*	
	*ER-positive*	0.256	0.089–0.740
	*HER2-positive*	4.902	1.484–16.195
	SUV-max tumor Δ	0.017	0.002–0.157

The AUC and confidence intervals of the models are shown in Table 5. At interim imaging, in the overall group, MRI appears to yield the strongest predictor of tumor response to NAC. When considering MRI as the reference, no other scenario yielded obviously superior performance.

**Table 5:** Area under the curve (AUC) and 95% confidence interval (95% CI) of all scenario models. The AUC of the interim scenarios were compared using scenario 1 (MRI only) as a reference. *Significant difference compared to scenario 1.

AUC (95% CI)	Overall	p-value	HER2-positive	p-value	ER-positive	p-value	Triple-negative	p-value
*Scenario 1*	0.894 (0.847–0.942)		0.735 (0.534–0.936)		0.742 (0.571–0.912)		0.855 (0.758–0.952)	
**versus**								
*Scenario 2*	0.894 (0.847–0.942)	0.250	0.735 (0.534–0.936)	0.250	0.742 (0.571–0.912)	0.250	0.855 (0.758–0.952)	0.250
*Scenario 3*	0.890 (0.843–0.936)	0.227	0.688 (0.508–0.868)	0.183	0.795 (0.674–0.917)	0.155	0.864 (0.768–0.961)	0.224
*Scenario 4*	0.892 (0.846–0.937)	0.238	0.708 (0.513–0.903)	0.213	0.818 (0.704–0.933)	0.117	0.868 (0.775–0.962)	0.213
*Scenario 5*	0.868 (0.816–0.920)	0.118	0.543 (0.362–0.724)	0.041*	0.791 (0.668–0.914)	0.162	0.844 (0.737–0.952)	0.220
*Scenario 6*	0.868 (0.816–0.920)	0.118	0.543 (0.362–0.724)	0.041*	0.791 (0.668–0.914)	0.162	0.844 (0.737–0.952)	0.220

**Fig 3:**
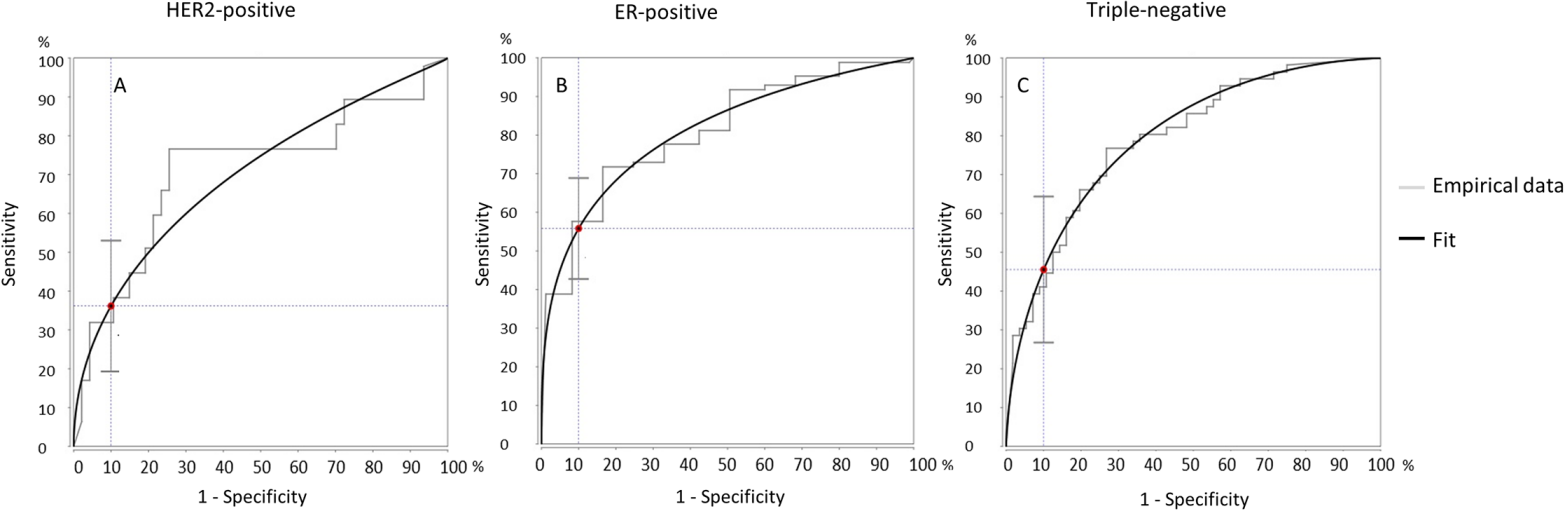
Fitted receiver operating characteristics (ROC) curves of the optimal imaging scenario for HER2-positive, ER-positive and Triple-negative tumors. A) ROC-curve of scenario 1 (MRI only) in HER2-positive tumors. B) ROC-curve of scenario 4 (MRI combined with PET/CT in incomplete responders) in ER-positive tumors. C) ROC-curve of scenario 1 (MRI only) in triple-negative tumors. For all ROC-curves an operating point at 90% specificity was selected to assess the corresponding sensitivity.

In Fig 3 the fitted ROC curves are shown of the optimal imaging scenario for HER2-positive, ER-positive and Triple-negative tumors. An operating point at 90% specificity was selected to assess the corresponding sensitivity, in other words, the probability of correctly predicting a non-pCRmic was determined under the condition that the probability to correctly predict a pCRmic is at least 90%.

For HER2-positive tumors, MRI was also the strongest predictor, performing significantly better than PET/CT. For this subtype, PET/CT was not found to have additional value. With scenario 1 (MRI only), at an operating point of 90% specificity, a sensitivity of 36.2% was achieved (Fig 3).

For ER-positive tumors, a favorable performance was seen from adding PET/CT to MRI, although no significant difference was seen to the MRI only scenario. Monitoring using PET/CT only also yielded favorable performance over that using MRI only. With scenario 4 (MRI combined with PET/CT in incomplete responders), at the 90% operating point, a sensitivity of 55.8% was achieved.

For triple-negative tumors only very small differences were seen between the different scenarios. With scenario 1 (MRI only), at a 90% specificity, a sensitivity of 45.5% was achieved.

## Discussion

The aim of this study was to explore guidelines in monitoring tumor response to NAC, taking breast cancer subtype into account and using different imaging scenarios: MRI only, PET/CT only, or a combination thereof. To pursue this aim, MRI and PET/CT were performed both prior to NAC as well as during NAC. Post-hoc analyses were performed to assess and compare the efficacy of scenarios. By systematically considering all combinations at different therapeutic windows in the clinical workflow, we found that the optimal imaging scenario depends considerably on breast cancer subtype.

For HER2-positive tumors, monitoring of tumor response to NAC was most accurately accomplished using MRI only. Approximately one third of the patients (36.2%) who did not achieve pCRmic could be identified at the cost of incorrectly assuming residual disease in 10% of the patients. PET/CT performed significantly less accurately (p = 0.04), while the combination of these techniques did not show obvious improvement.

For triple-negative tumors, monitoring of response was also most accurately accomplished using MRI only. Approximately half the number of patients (45%) who did not achieve pCRmic could be identified, at the cost of incorrectly assuming residual disease in 10% of patients. For these tumors, little difference was seen between the performance of PET/CT and MRI. This suggests that PET/CT is an appropriate alternative to MRI for patients with triple-negative tumors with contraindications for MRI.

For ER-positive tumors, PET/CT showed slightly favorable performance compared to MRI, and results suggest that response monitoring of ER-positive tumors may be optimized by combining MRI with PET/CT. Using this latter scenario, half the number of patients without pCRmic could be identified while residual disease was incorrectly assumed in 10% of the patients. However, even though relatively large numbers of ER-positive tumor patients were included, no significant differences were found between the scenarios.

It is widely recognized that the different breast cancer subtypes prompt different treatments, variant responses to treatment, and that they are linked to different prognosis. As seen seen in this current study, different subtypes are also linked to different optimal imaging scenarios.

In prior studies, the strictest definition of pCRmic (i.e., no residual invasive disease in the breast or axilla: ypT0 ypN0) was found to be associated with increased disease-free and overall survival in subgroups of patients, mostly so in HER2-positive and triple-negative tumors [28]. However, in this study we chose pCRmic: complete absence of tumor cells or presence of only a small number of scattered invasive cells in the resection specimen (ypTmic) as the endpoint of this study was not survival, but rather assessment of sensitivity and specificity of imaging for response monitoring. Second, the association between ypT0 ypN0 and survival has not been shown for luminal A tumors, which comprise the largest subgroup of breast cancers, including the ER-positive tumors. Thirdly, as comparable numbers of ypT0 ypN0 were found compared to the results of other studies: 5–10% of ER-positive tumors, 20–30% of triple-negative tumors, and 30–65% of HER2-positive tumors (treated with a combinations of NAC and trastuzumab) [28–31], there was insufficient power to use ypT0 ypN0 as endpoint in ER-positive tumors In this study. Using ypTmic, tumor response was found in 11/87 ER-positive tumors (12.6%), providing sufficient power to assess the sensitivity of MRI, PET/CT and combination thereof. In future studies, other study endpoints could be considered, such as the possibility for breast conserving surgery following NAC, as improvement of surgical options is still one of the major reasons to consider NAC [32].

Future studies could also consider the inclusion of diffusion-weighted MR imaging (DW-MRI), as promising results have been shown in the use of DWI to monitor early tumor response of breast cancers to NAC [33]. For PET/CT imaging, the SUV-max of tumors and lymph nodes were evaluated because these are most commonly assessed in clinical practice. However, future study could consider other imaging characteristics such as the total lesion glycolysis [34]. Also, the use of ^18^F-fluoroestradiol or ^89^ZR-trastuzumab could be considered for PET/CT response monitoring in certain breast cancer subtypes [35,36]. Future study could also consider whether subtle changes in breast tissue, for example due to age-related changes in breast structure and density, is of influence to the sensitivity of MRI and PET/CT imaging in the current study setting. Currently, this was not assessed due to limited patient numbers within the different subgroups. For the MRI and PET/CT parameters we did not address inter- or intra-observer variation. The parameters were assessed under realistic clinical conditions to obtain their value in routine clinical practice. However, future studies could focus on automated techniques to extract complementary information from MRI and PET/CT to monitor breast cancer response [37].

## Conclusions

For imaging response of breast cancer to neoadjuvant chemotherapy, MRI was found optimal to monitor response for HER2-positive and triple-negative tumors. For HER2-positive tumors, MRI has an advantage over PET/CT imaging as well as over combined techniques. However, for triple-negative tumors, PET/CT is an appropriate alternative in patients with contraindications for MRI. For ER-positive tumors, PET/CT shows favorable performance over MRI, and combining PET/CT with MRI could provide optimal response monitoring. However, even though relatively large numbers of ER-positive tumor patients were included, significant differences could not yet be shown.

## Supporting information

S1 Table Supporting information table containing age, breast cancer subtype, pathological complete response (pCRmic), change (Δ) of maximum standardized uptake value (SUV-max), and change of largest tumor diameter (LD) on initial and late enhancement on MRI. (DOCX)

## References

[1] Fisher B, Brown A, Mamounas E, Wieand S, Robidoux A, Margolese RG, et al Effect of preoperative chemotherapy on local-regional disease in women with operable breast cancer: findings from National Surgical Adjuvant Breast and Bowel Project B-18. J Clin Oncol 1997 7;15(7):2483–93. doi: 10.1200/JCO.1997.15.7.2483 9215816 10.1200/JCO.1997.15.7.2483

[2] Mieog JS, van der Hage JA, van de Velde CJ. Neoadjuvant chemotherapy for operable breast cancer. Br J Surg 2007 10;94(10):1189–200. doi: 10.1002/bjs.5894 17701939 10.1002/bjs.5894

[3] Fitzal F, Riedl O, Mittlbock M, Dubsky P, Bartsch R, Steger G, et al Oncologic safety of breast conserving surgery after tumour downsizing by neoadjuvant therapy: a retrospective single centre cohort study. Breast Cancer Res Treat 2011 5;127(1):121–8. doi: 10.1007/s10549-010-1164-9 20848185 10.1007/s10549-010-1164-9

[4] Donker M, Straver ME, Wesseling J, Loo CE, Schot M, Drukker CA, et al Marking Axillary Lymph Nodes With Radioactive Iodine Seeds for Axillary Staging After Neoadjuvant Systemic Treatment in Breast Cancer Patients: The MARI Procedure. Ann Surg 2014 4 16. 10.1097/SLA.0000000000000558 24743607

[5] Li JW, Mo M, Yu KD, Chen CM, Hu Z, Hou YF, et al ER-Poor and HER2-Positive: A Potential Subtype of Breast Cancer to Avoid Axillary Dissection in Node Positive Patients after Neoadjuvant Chemo-Trastuzumab Therapy. PLoS One 2014;9(12):e114646 doi: 10.1371/journal.pone.0114646 25504233 10.1371/journal.pone.0114646 PMC4263615

[6] Straver ME, Rutgers EJ, Russell NS, Oldenburg HS, Rodenhuis S, Wesseling J, et al Towards rational axillary treatment in relation to neoadjuvant therapy in breast cancer. Eur J Cancer 2009 9;45(13):2284–92. doi: 10.1016/j.ejca.2009.04.029 19464164 10.1016/j.ejca.2009.04.029

[7] Schwartz GF, Hortobagyi GN, Masood S, Palazzo J, Holland R, Page D. Proceedings of the consensus conference on neoadjuvant chemotherapy in carcinoma of the breast, April 26–28, 2003, Philadelphia, PA. Hum Pathol 2004 Jul;35(7):781–4. 10.1016/j.humpath.2004.02.006 15257539

[8] Loo CE, Teertstra HJ, Rodenhuis S, van de Vijver MJ, Hannemann J, Muller SH, et al Dynamic contrast-enhanced MRI for prediction of breast cancer response to neoadjuvant chemotherapy: initial results. AJR Am J Roentgenol 2008 11;191(5):1331–8. doi: 10.2214/AJR.07.3567 18941065 10.2214/AJR.07.3567

[9] Hylton NM, Blume JD, Bernreuter WK, Pisano ED, Rosen MA, Morris EA, et al Locally advanced breast cancer: MR imaging for prediction of response to neoadjuvant chemotherapy—results from ACRIN 6657/I-SPY TRIAL. Radiology 2012 6;263(3):663–72. doi: 10.1148/radiol.12110748 22623692 10.1148/radiol.12110748 PMC3359517

[10] Fangberget A, Nilsen LB, Hole KH, Holmen MM, Engebraaten O, Naume B, et al Neoadjuvant chemotherapy in breast cancer-response evaluation and prediction of response to treatment using dynamic contrast-enhanced and diffusion-weighted MR imaging. Eur Radiol 2011 6;21(6):1188–99. doi: 10.1007/s00330-010-2020-3 21127880 10.1007/s00330-010-2020-3 PMC3088808

[11] Kuhl CK. Current status of breast MR imaging. Part 2. Clinical applications. Radiology 2007 9;244(3):672–91. doi: 10.1148/radiol.2443051661 17709824 10.1148/radiol.2443051661

[12] Loo CE, Straver ME, Rodenhuis S, Muller SH, Wesseling J, Vrancken Peeters MJ, et al Magnetic resonance imaging response monitoring of breast cancer during neoadjuvant chemotherapy: relevance of breast cancer subtype. J Clin Oncol 2011 2 20;29(6):660–6. doi: 10.1200/JCO.2010.31.1258 21220595 10.1200/JCO.2010.31.1258

[13] Groheux D, Espie M, Giacchetti S, Hindie E. Performance of FDG PET/CT in the clinical management of breast cancer. Radiology 2013 2;266(2):388–405. doi: 10.1148/radiol.12110853 23220901 10.1148/radiol.12110853

[14] Koolen BB, Vrancken Peeters MJ, Aukema TS, Vogel WV, Oldenburg HS, van der Hage JA, et al 18F-FDG PET/CT as a staging procedure in primary stage II and III breast cancer: comparison with conventional imaging techniques. Breast Cancer Res Treat 2012 1;131(1):117–26. doi: 10.1007/s10549-011-1767-9 21935602 10.1007/s10549-011-1767-9

[15] Duch J, Fuster D, Munoz M, Fernandez PL, Paredes P, Fontanillas M, et al 18F-FDG PET/CT for early prediction of response to neoadjuvant chemotherapy in breast cancer. Eur J Nucl Med Mol Imaging 2009 10;36(10):1551–7. doi: 10.1007/s00259-009-1116-y 19326117 10.1007/s00259-009-1116-y

[16] Kumar A, Kumar R, Seenu V, Gupta SD, Chawla M, Malhotra A, et al The role of 18F-FDG PET/CT in evaluation of early response to neoadjuvant chemotherapy in patients with locally advanced breast cancer. Eur Radiol 2009 6;19(6):1347–57. doi: 10.1007/s00330-009-1303-z 19214522 10.1007/s00330-009-1303-z

[17] Koolen BB, Pengel KE, Wesseling J, Vogel WV, Vrancken Peeters MJ, Vincent AD, et al FDG PET/CT during neoadjuvant chemotherapy may predict response in ER-positive/HER2-negative and triple negative, but not in HER2-positive breast cancer. Breast 2013 10;22(5):691–7. doi: 10.1016/j.breast.2012.12.020 23414930 10.1016/j.breast.2012.12.020

[18] Pengel KE, Koolen BB, Loo CE, Vogel WV, Wesseling J, Lips EH, et al Combined use of (18)F-FDG PET/CT and MRI for response monitoring of breast cancer during neoadjuvant chemotherapy. Eur J Nucl Med Mol Imaging 2014 8;41(8):1515–24. doi: 10.1007/s00259-014-2770-2 24777490 10.1007/s00259-014-2770-2

[19] Sonke GS, Mandjes IA, Holtkamp MJ, Schot M, van WE, Wesseling J, et al Paclitaxel, carboplatin, and trastuzumab in a neo-adjuvant regimen for HER2-positive breast cancer. Breast J 2013 7;19(4):419–26. doi: 10.1111/tbj.12124 23682812 10.1111/tbj.12124

[20] Rigter LS, Loo CE, Linn SC, Sonke GS, van WE, Lips EH, et al Neoadjuvant chemotherapy adaptation and serial MRI response monitoring in ER-positive HER2-negative breast cancer. Br J Cancer 2013 12 10;109(12):2965–72. doi: 10.1038/bjc.2013.661 24149178 10.1038/bjc.2013.661 PMC3859944

[21] Koolen BB, Pengel KE, Wesseling J, Vogel WV, Vrancken Peeters MJ, Vincent AD, et al Sequential (18)F-FDG PET/CT for early prediction of complete pathological response in breast and axilla during neoadjuvant chemotherapy. Eur J Nucl Med Mol Imaging 2014 1;41(1):32–40. doi: 10.1007/s00259-013-2515-7 23929431 10.1007/s00259-013-2515-7

[22] Gilhuijs KG, Deurloo EE, Muller SH, Peterse JL, Schultze Kool LJ. Breast MR imaging in women at increased lifetime risk of breast cancer: clinical system for computerized assessment of breast lesions initial results. Radiology 2002 12;225(3):907–16. doi: 10.1148/radiol.2253011582 12461278 10.1148/radiol.2253011582

[23] Kuhl CK, Mielcareck P, Klaschik S, Leutner C, Wardelmann E, Gieseke J, et al Dynamic breast MR imaging: are signal intensity time course data useful for differential diagnosis of enhancing lesions? Radiology 1999 4;211(1):101–10. doi: 10.1148/radiology.211.1.r99ap38101 10189459 10.1148/radiology.211.1.r99ap38101

[24] Aukema TS, Vogel WV, Hoefnagel CA, Valdes Olmos RA. Prevention of brown adipose tissue activation in 18F-FDG PET/CT of breast cancer patients receiving neoadjuvant systemic therapy. J Nucl Med Technol 2010 3;38(1):24–7. doi: 10.2967/jnmt.109.065557 20159933 10.2967/jnmt.109.065557

[25] Sataloff DM, Mason BA, Prestipino AJ, Seinige UL, Lieber CP, Baloch Z. Pathologic response to induction chemotherapy in locally advanced carcinoma of the breast: a determinant of outcome. J Am Coll Surg 1995 3;180(3):297–306. 7874340

[26] DeLong ER, Delong DM, Clarke-Pearson DL. Comparing the areas under two or more correlated receiver operating characteristic curves: a nonparametric approach. Biometrics 1988 9;44(3):837–45. 3203132

[27] Pesce LL, Metz CE. Reliable and computationally efficient maximum-likelihood estimation of "proper" binormal ROC curves. Acad Radiol 2007 7;14(7):814–29. doi: 10.1016/j.acra.2007.03.012 17574132 10.1016/j.acra.2007.03.012 PMC2693394

[28] von Minckwitz G, Untch M, Blohmer JU, Costa SD, Eidtmann H, Fasching PA, et al Definition and impact of pathologic complete response on prognosis after neoadjuvant chemotherapy in various intrinsic breast cancer subtypes. Journal of Clinical Oncology 2012;JCO-2011. 10.1200/JCO.2011.38.8595 22508812

[29] Untch M, Fasching PA, Konecny GE, Hasmuller S, Lebeau A, Kreienberg R, et al Pathologic complete response after neoadjuvant chemotherapy plus trastuzumab predicts favorable survival in human epidermal growth factor receptor 2-overexpressing breast cancer: results from the TECHNO trial of the AGO and GBG study groups. J Clin Oncol 2011 9 1;29(25):3351–7. doi: 10.1200/JCO.2010.31.4930 21788566 10.1200/JCO.2010.31.4930

[30] Buzdar AU, Ibrahim NK, Francis D, Booser DJ, Thomas ES, Theriault RL, et al Significantly higher pathologic complete remission rate after neoadjuvant therapy with trastuzumab, paclitaxel, and epirubicin chemotherapy: results of a randomized trial in human epidermal growth factor receptor 2-positive operable breast cancer. J Clin Oncol 2005 6 1;23(16):3676–85. doi: 10.1200/JCO.2005.07.032 15738535 10.1200/JCO.2005.07.032

[31] Liedtke C, Mazouni C, Hess KR, Andre F, Tordai A, Mejia JA, et al Response to neoadjuvant therapy and long-term survival in patients with triple-negative breast cancer. J Clin Oncol 2008 3 10;26(8):1275–81. doi: 10.1200/JCO.2007.14.4147 18250347 10.1200/JCO.2007.14.4147

[32] Kaufmann M, von MG, Mamounas EP, Cameron D, Carey LA, Cristofanilli M, et al Recommendations from an international consensus conference on the current status and future of neoadjuvant systemic therapy in primary breast cancer. Ann Surg Oncol 2012 5;19(5):1508–16. doi: 10.1245/s10434-011-2108-2 22193884 10.1245/s10434-011-2108-2

[33] Sharma U, Danishad KK, Seenu V, Jagannathan NR. Longitudinal study of the assessment by MRI and diffusion-weighted imaging of tumor response in patients with locally advanced breast cancer undergoing neoadjuvant chemotherapy. NMR Biomed 2009 1;22(1):104–13. doi: 10.1002/nbm.1245 18384182 10.1002/nbm.1245

[34] Groheux D, Majdoub M, Sanna A, de CP, Hindie E, Giacchetti S, et al Early Metabolic Response to Neoadjuvant Treatment: FDG PET/CT Criteria according to Breast Cancer Subtype. Radiology 2015 4 27;141638. 10.1148/radiol.2015141638 25915099

[35] Linden HM, Stekhova SA, Link JM, Gralow JR, Livingston RB, Ellis GK, et al Quantitative fluoroestradiol positron emission tomography imaging predicts response to endocrine treatment in breast cancer. J Clin Oncol 2006 6 20;24(18):2793–9. doi: 10.1200/JCO.2005.04.3810 16682724 10.1200/JCO.2005.04.3810

[36] Dijkers EC, Oude Munnink TH, Kosterink JG, Brouwers AH, Jager PL, de Jong JR, et al Biodistribution of 89Zr-trastuzumab and PET imaging of HER2-positive lesions in patients with metastatic breast cancer. Clin Pharmacol Ther 2010 5;87(5):586–92. doi: 10.1038/clpt.2010.12 20357763 10.1038/clpt.2010.12

[37] Dmitriev ID, Loo CE, Vogel WV, Pengel KE, Gilhuijs KG. Fully automated deformable registration of breast DCE-MRI and PET/CT. Phys Med Biol 2013 2 21;58(4):1221–33. doi: 10.1088/0031-9155/58/4/1221 23369926 10.1088/0031-9155/58/4/1221

