# Supplementary material for: Monitoring tumor response to neoadjuvant chemotherapy using MRI and 18F-FDG PET/CT in breast cancer subtypes
Source: PLoS One. 2017 May 22;12(5):e0176782. doi: 10.1371/journal.pone.0176782 (PMC5439668; doi:10.1371/journal.pone.0176782)
Supplement: S1 Table — (DOCX) [file pone.0176782.s001.docx]

**Supporting information file**

| Patient | Age | Her2-pos (1) /  ER-pos Her2-neg (2) / Triple neg (3) | pCRmic (1)/  Non-pCRmic (0) | SUV-max tumor ∆ | SUV max LN ∆ | MRI LD initial ∆ | MRI LD late ∆ |
| --- | --- | --- | --- | --- | --- | --- | --- |
| 1 | 58,4 | 1 | 1 | -0,5 | -0,1 | -1 | -1 |
| 2 | 68,1 | 3 | 0 | -0,5 | -0,6 | -0,19 | -0,1 |
| 3 | 60 | 2 | 0 | -0,2 | -0,7 | -0,23 | -1 |
| 4 | 49,5 | 1 | 1 | -0,6 | -0,7 | -1 | -1 |
| 5 | 43,3 | 2 | 0 | -0,3 | -1 | -0,18 | -1 |
| 6 | 67,9 | 1 | 0 | -0,6 | N/A | 0,02 | -0,8 |
| 7 | 62,1 | 2 | 0 | -1 | N/A | -0,14 | -1 |
| 8 | 57 | 2 | 0 | 0,2 | N/A | -0,06 | -0,1 |
| 9 | 49,6 | 2 | 0 | -1 | -0,6 | -0,2 | -0,4 |
| 10 | 43,7 | 1 | 1 | -0,3 | -0,7 | -1 | -1 |
| 11 | 61,3 | 2 | 0 | -0,6 | -0,7 | -1 | -1 |
| 12 | 41,1 | 2 | 1 | -0,9 | -0,7 | -0,71 | -0,9 |
| 13 | 61 | 2 | 0 | -0,5 | N/A | -0,17 | -0,2 |
| 14 | 38,9 | 3 | 0 | -0,6 | N/A | -0,7 | -0,6 |
| 15 | 54 | 2 | 0 | -0,4 | -1 | -0,2 | -1 |
| 16 | 43,6 | 1 | 0 | -0,7 | N/A | -0,5 | -0,5 |
| 17 | 50,3 | 2 | 0 | 0,6 | N/A | -0,1 | 0 |
| 18 | 42,4 | 2 | 0 | -0,4 | N/A | -0,06 | -0,1 |
| 19 | 67,8 | 3 | 1 | -0,2 | N/A | -0,19 | -1 |
| 20 | 29,8 | 3 | 1 | -0,8 | -1 | -1 | -1 |
| 21 | 38,2 | 2 | 0 | -0,4 | -0,7 | -0,29 | -0,2 |
| 22 | 40,3 | 1 | 0 | -0,7 | N/A | -0,2 | -0,3 |
| 23 | 66,7 | 3 | 0 | -0,5 | -0,5 | -0,21 | -0,4 |
| 24 | 54,9 | 3 | 0 | -0,3 | -0,8 | -0,04 | 0 |
| 25 | 60,5 | 1 | 1 | -0,5 | -0,6 | -0,52 | -1 |
| 26 | 46,8 | 1 | 1 | -0,4 | -1 | -0,67 | -1 |
| 27 | 40,9 | 2 | 0 | -0,8 | -0,9 | -0,36 | -1 |
| 28 | 51,9 | 2 | 0 | 0 | -0,4 | -0,14 | -0,2 |
| 29 | 58,8 | 1 | 1 | -0,6 | -0,4 | -0,56 | -0,6 |
| 30 | 30,9 | 2 | 1 | -0,7 | -0,9 | 0,03 | -0,3 |
| 31 | 36,8 | 1 | 0 | -0,8 | -1 | -0,37 | -0,5 |
| 32 | 50,4 | 1 | 1 | -0,5 | -0,5 | -1 | -1 |
| 33 | 52,1 | 2 | 0 | -0,3 | -0,2 | -0,15 | -0,2 |
| 34 | 54,7 | 3 | 1 | -0,8 | -0,9 | -0,64 | -1 |
| 35 | 48,1 | 2 | 0 | -0,7 | -0,6 | -0,09 | -0,4 |
| 36 | 65,2 | 3 | 1 | -0,5 | -0,9 | -0,51 | -1 |
| 37 | 36,9 | 2 | 0 | -0,2 | -0,3 | 0,27 | 0 |
| 38 | 54,6 | 1 | 1 | -0,7 | -0,7 | -0,36 | -0,3 |
| 39 | 31,2 | 3 | 1 | -0,9 | N/A | -1 | -1 |
| 40 | 47,6 | 3 | 1 | -0,7 | N/A | -0,58 | -0,8 |
| 41 | 45,1 | 2 | 0 | -0,7 | N/A | -0,23 | -0,1 |
| 42 | 52,5 | 1 | 1 | -0,4 | -1 | -1 | -1 |
| 43 | 48,7 | 2 | 0 | -0,6 | -0,4 | -0,05 | -0,3 |
| 44 | 64,7 | 3 | 0 | -0,4 | N/A | -0,3 | -0,3 |
| 45 | 60,3 | 2 | 0 | -0,4 | -0,4 | -0,46 | -1 |
| 46 | 34,3 | 1 | 0 | -0,6 | -0,8 | -0,41 | -0,5 |
| 47 | 37 | 1 | 0 | -0,4 | -1 | -1 | -1 |
| 48 | 42,8 | 3 | 1 | -0,4 | -0,4 | -0,4 | -0,4 |
| 49 | 38,1 | 1 | 1 | -0,8 | -0,7 | -0,5 | -1 |
| 50 | 36,5 | 2 | 0 | -0,5 | -1 | -0,17 | 0 |
| 51 | 51,6 | 2 | 0 | -0,4 | -0,2 | -0,13 | -0,1 |
| 52 | 56,1 | 3 | 0 | -0,4 | -1 | -0,28 | -0,2 |
| 53 | 43,7 | 2 | 0 | -0,7 | -0,6 | -0,12 | -0,4 |
| 54 | 48,5 | 1 | 1 | -0,9 | -1 | -0,73 | -0,7 |
| 55 | 42,7 | 3 | 1 | -0,7 | N/A | -0,09 | -0,2 |
| 56 | 39,6 | 2 | 0 | -0,3 | -0,5 | -0,2 | -0,2 |
| 57 | 45,9 | 2 | 0 | -0,3 | N/A | 0 | 0 |
| 58 | 32 | 3 | 1 | -0,7 | -0,8 | -0,1 | -0,2 |
| 59 | 45,6 | 2 | 1 | -0,8 | -1 | -0,08 | -0,1 |
| 60 | 61,2 | 2 | 0 | -0,6 | -1 | -0,17 | -0,3 |
| 61 | 51 | 1 | 0 | -0,8 | -0,5 | -1 | -1 |
| 62 | 45,5 | 2 | 0 | -0,3 | -0,4 | -0,02 | -0,4 |
| 63 | 42,8 | 2 | 0 | -0,7 | N/A | -0,59 | -0,6 |
| 64 | 25,8 | 3 | 1 | -0,9 | -0,9 | -1 | -1 |
| 65 | 50,5 | 1 | 0 | -0,8 | -0,9 | -1 | -1 |
| 66 | 50,5 | 1 | 1 | -0,7 | -1 | -0,88 | -0,9 |
| 67 | 57,1 | 1 | 1 | -0,6 | -1 | -0,79 | -1 |
| 68 | 50,6 | 2 | 1 | -0,7 | -1 | -0,43 | -1 |
| 69 | 40,2 | 1 | 1 | -0,3 | -1 | -1 | -1 |
| 70 | 29,7 | 3 | 0 | -0,5 | N/A | -0,52 | -0,4 |
| 71 | 28,5 | 2 | 0 | -0,4 | -0,7 | -0,32 | -0,3 |
| 72 | 28,6 | 1 | 1 | -0,6 | 0 | -1 | -1 |
| 73 | 39,6 | 1 | 1 | -0,7 | -1 | -1 | -1 |
| 74 | 37,8 | 3 | 1 | -0,5 | N/A | -0,25 | -0,1 |
| 75 | 59,7 | 1 | 0 | -0,8 | -0,6 | -0,63 | -0,6 |
| 76 | 44 | 3 | 0 | -0,2 | -0,3 | 0,13 | 0,1 |
| 77 | 31,4 | 2 | 0 | 0,2 | -0,1 | 0,16 | 0,2 |
| 78 | 52,3 | 1 | 1 | -0,2 | -1 | -1 | -1 |
| 79 | 40,6 | 2 | 0 | -0,2 | N/A | -0,24 | -0,3 |
| 80 | 49,9 | 2 | 0 | -0,3 | -1 | -0,4 | -1 |
| 81 | 30,3 | 1 | 1 | -0,7 | -1 | -1 | -1 |
| 82 | 44,9 | 2 | 0 | -0,4 | N/A | -0,73 | -0,5 |
| 83 | 48,5 | 2 | 0 | -0,4 | N/A | -0,14 | -0,2 |
| 84 | 47,9 | 3 | 1 | -0,8 | -1 | -0,62 | -0,6 |
| 85 | 29,4 | 3 | 1 | -0,8 | -1 | -1 | -1 |
| 86 | 50,5 | 3 | 0 | -0,5 | -0,3 | -0,02 | 0,2 |
| 87 | 63,8 | 2 | 0 | 0,1 | 0,2 | 0,06 | 0,1 |
| 88 | 49,7 | 3 | 1 | -0,7 | -0,5 | -0,48 | -1 |
| 89 | 47,8 | 2 | 1 | -0,5 | -0,6 | 0 | -0,1 |
| 90 | 52,4 | 1 | 1 | -0,8 | -0,9 | -1 | -1 |
| 91 | 26,4 | 3 | 1 | -0,7 | -0,7 | -1 | -1 |
| 92 | 60,4 | 2 | 0 | -0,3 | N/A | -0,1 | -0,4 |
| 93 | 54,3 | 2 | 1 | -0,5 | -1 | -1 | -1 |
| 94 | 39,5 | 3 | 1 | -0,8 | -0,8 | -0,43 | -1 |
| 95 | 43,6 | 1 | 1 | -0,8 | -0,8 | -1 | -1 |
| 96 | 41,1 | 3 | 1 | -0,7 | N/A | -0,61 | -0,6 |
| 97 | 51,1 | 3 | 0 | -0,4 | -0,5 | -0,18 | -0,2 |
| 98 | 35,8 | 3 | 1 | -0,7 | -0,9 | -1 | -1 |
| 99 | 49 | 2 | 0 | -0,6 | -0,7 | -0,26 | -1 |
| 100 | 41,7 | 2 | 0 | -0,2 | N/A | -0,47 | -0,2 |
| 101 | 37,8 | 2 | 0 | -0,2 | -0,5 | -0,31 | -0,3 |
| 102 | 59,9 | 2 | 0 | -0,2 | -0,4 | -0,36 | -0,7 |
| 103 | 52,6 | 1 | 1 | -0,8 | -0,6 | -1 | -1 |
| 104 | 32,8 | 3 | 1 | -0,8 | -0,8 | -0,49 | -1 |
| 105 | 62,1 | 2 | 0 | -0,6 | -0,7 | -0,2 | -0,3 |
| 106 | 39,1 | 2 | 0 | -0,5 | -0,5 | -0,23 | -0,2 |
| 107 | 55,3 | 1 | 1 | -0,5 | -0,7 | -0,7 | -1 |
| 108 | 33,1 | 3 | 0 | -0,5 | N/A | -0,43 | -0,4 |
| 109 | 44,2 | 3 | 1 | -0,4 | -0,3 | -0,16 | -0,1 |
| 110 | 53,9 | 2 | 0 | -0,4 | -0,7 | -0,56 | -0,5 |
| 111 | 51,7 | 2 | 0 | -0,8 | -0,7 | -0,42 | -0,5 |
| 112 | 34,9 | 3 | 1 | -0,8 | N/A | -0,69 | -0,7 |
| 113 | 63,5 | 2 | 0 | 0,1 | 0,1 | -0,04 | 0 |
| 114 | 53,3 | 2 | 0 | -0,4 | N/A | -0,13 | -0,2 |
| 115 | 64,1 | 3 | 0 | -0,6 | -0,8 | -0,54 | -0,7 |
| 116 | 59,6 | 2 | 0 | -0,6 | -0,8 | -0,19 | -1 |
| 117 | 46,2 | 2 | 0 | -0,6 | -0,3 | -0,44 | -1 |
| 118 | 55,8 | 1 | 1 | -0,7 | -0,5 | -0,38 | -1 |
| 119 | 46,7 | 2 | 0 | -0,6 | -0,7 | 0,01 | -0,1 |
| 120 | 50,6 | 2 | 1 | -0,7 | -0,7 | -0,09 | -0,5 |
| 121 | 28 | 3 | 1 | -0,9 | -0,9 | -1 | -1 |
| 122 | 38,1 | 2 | 0 | -0,4 | -0,8 | -0,3 | -1 |
| 123 | 26,1 | 3 | 1 | -0,9 | -1 | -1 | -1 |
| 124 | 43,9 | 3 | 0 | -0,7 | -0,5 | -0,56 | -1 |
| 125 | 27,1 | 3 | 1 | -0,9 | 0,1 | -0,67 | -1 |
| 126 | 40,2 | 3 | 1 | -0,6 | N/A | -1 | -1 |
| 127 | 62,8 | 2 | 0 | -0,3 | -0,3 | -0,13 | -0,1 |
| 128 | 42,9 | 2 | 1 | -0,7 | -0,8 | -0,3 | -1 |
| 129 | 45,7 | 2 | 0 | -0,5 | N/A | -0,33 | -0,3 |
| 130 | 54,6 | 3 | 0 | -0,1 | -0,2 | -0,03 | -0,1 |
| 131 | 38,8 | 3 | 1 | -0,7 | N/A | -0,58 | -0,8 |
| 132 | 39,8 | 1 | 1 | -0,8 | -0,3 | -1 | -1 |
| 133 | 47,7 | 2 | 0 | -0,8 | N/A | 0 | 0 |
| 134 | 34,7 | 2 | 0 | -1 | N/A | -0,59 | -1 |
| 135 | 49,4 | 3 | 0 | -1 | -0,7 | -0,05 | -0,1 |
| 136 | 64,3 | 3 | 0 | -0,5 | -0,4 | -0,16 | -0,3 |
| 137 | 59 | 3 | 0 | -0,6 | N/A | -0,41 | -0,5 |
| 138 | 46,8 | 2 | 0 | -0,4 | -0,4 | -0,21 | -0,8 |
| 139 | 25,8 | 1 | 1 | -0,7 | -1 | -0,23 | -1 |
| 140 | 48,7 | 2 | 0 | -0,3 | N/A | -0,36 | -0,4 |
| 141 | 40,9 | 2 | 1 | -0,4 | N/A | -0,81 | -0,7 |
| 142 | 48,4 | 2 | 0 | -0,4 | -0,5 | -0,2 | -0,2 |
| 143 | 36,6 | 2 | 0 | -0,6 | N/A | -0,04 | -0,2 |
| 144 | 33,9 | 3 | 1 | -0,8 | -0,7 | -0,43 | -0,4 |
| 145 | 28,9 | 3 | 0 | -0,7 | -0,9 | -0,63 | -0,6 |
| 146 | 43,7 | 3 | 1 | -0,6 | N/A | -0,24 | -0,6 |
| 147 | 51,8 | 3 | 0 | -0,2 | -1 | -0,43 | -0,4 |
| 148 | 44,8 | 3 | 0 | -0,2 | N/A | -0,32 | -0,9 |
| 149 | 73,1 | 1 | 1 | -0,8 | -1 | -0,57 | -0,6 |
| 150 | 65,4 | 2 | 0 | -0,5 | -0,6 | -0,15 | -0,3 |
| 151 | 46 | 2 | 0 | -0,4 | -1 | -0,1 | -1 |
| 152 | 51 | 3 | 1 | -0,7 | -1 | -0,83 | -0,9 |
| 153 | 51,3 | 2 | 0 | -0,6 | N/A | -0,21 | -0,3 |
| 154 | 53,7 | 3 | 0 | -0,6 | -0,8 | 0,08 | 0 |
| 155 | 59,8 | 2 | 0 | -0,4 | -0,3 | -0,14 | -0,1 |
| 156 | 48,4 | 2 | 1 | -0,5 | -0,7 | -0,18 | -0,8 |
| 157 | 50,9 | 2 | 0 | -0,3 | -1 | -0,01 | 0 |
| 158 | 48,5 | 2 | 0 | -0,4 | N/A | -0,34 | -0,7 |
| 159 | 39,7 | 2 | 0 | 0,4 | 0,1 | -0,17 | -0,3 |
| 160 | 35,4 | 3 | 1 | -0,5 | N/A | -1 | -1 |
| 161 | 46,6 | 2 | 0 | -0,2 | N/A | 0,05 | 0 |
| 162 | 47,6 | 2 | 0 | -0,5 | -1 | -0,33 | -0,2 |
| 163 | 54,1 | 2 | 0 | -0,6 | -0,5 | -0,15 | -0,1 |
| 164 | 42,2 | 2 | 0 | -0,5 | N/A | -0,32 | -1 |
| 165 | 50,2 | 3 | 0 | -0,5 | -0,5 | -0,67 | -0,7 |
| 166 | 51 | 1 | 0 | 0,4 | 0,1 | -0,03 | -0,1 |
| 167 | 38,2 | 1 | 1 | -0,7 | -0,6 | -1 | -1 |
| 168 | 64 | 3 | 0 | -0,1 | N/A | 0 | 0 |
| 169 | 46,3 | 1 | 1 | -0,8 | -1 | -0,92 | -1 |
| 170 | 70,2 | 1 | 1 | -0,8 | N/A | -1 | -1 |
| 171 | 46,6 | 2 | 0 | -0,1 | -1 | -0,12 | -0,1 |
| 172 | 65,1 | 2 | 0 | -0,2 | N/A | -0,39 | -0,4 |
| 173 | 35,7 | 1 | 1 | -0,5 | -1 | -0,67 | -0,6 |
| 174 | 64,9 | 2 | 0 | -0,3 | 0,1 | -0,19 | -0,1 |
| 175 | 62,9 | 2 | 1 | -0,9 | -0,9 | -1 | -1 |
| 176 | 27,4 | 1 | 1 | -1 | N/A | -0,45 | -1 |
| 177 | 57,5 | 2 | 0 | -0,4 | N/A | 0,02 | -0,4 |
| 178 | 42,2 | 2 | 0 | -0,4 | -0,6 | -0,11 | -0,1 |
| 179 | 49,9 | 1 | 0 | -0,6 | N/A | -0,11 | -1 |
| 180 | 51,4 | 1 | 1 | -0,7 | 0,6 | -0,3 | -1 |
| 181 | 44,4 | 1 | 1 | -0,8 | -0,5 | -0,45 | -0,4 |
| 182 | 25,4 | 1 | 1 | -0,8 | -0,9 | -0,26 | -0,6 |
| 183 | 60,7 | 1 | 1 | -0,8 | -0,8 | -1 | -1 |
| 184 | 66,5 | 3 | 0 | -0,6 | N/A | -0,31 | -0,5 |
| 185 | 50,3 | 3 | 1 | -0,7 | -0,8 | -0,45 | -0,5 |
| 186 | 44,9 | 2 | 0 | -0,5 | -0,4 | -0,28 | -0,3 |
| 187 | 48,5 | 2 | 0 | -0,5 | -0,1 | -0,36 | -0,6 |
| 188 | 51,4 | 2 | 0 | -0,6 | N/A | -0,38 | -1 |

**Supporting information file** containing age, breast cancer subtype, pathological complete response (pCRmic), change (∆) of maximum standardized uptake value (SUV-max), and change of largest tumor diameter (LD) on initial and late enhancement on MRI.
